# Cyclooxygenase Isoform Exchange Blocks Brain-Mediated Inflammatory Symptoms

**DOI:** 10.1371/journal.pone.0166153

**Published:** 2016-11-18

**Authors:** Daniel Björk Wilhelms, Elahe Mirrasekhian, Joanna Zajdel, Anand Kumar Singh, David Engblom

**Affiliations:** 1 Department of Clinical and Experimental Medicine, Linköping University, Linköping, Sweden; 2 Division of Drug Research/Emergency Medicine, Department of Medical and Health Sciences, Linköping University, Linköping, Sweden; 3 Department of Emergency Medicine, Linköping University Hospital, Linköping, Sweden; Universidade de Sao Paulo, BRAZIL

## Abstract

Cyclooxygenase-2 (COX-2) is the main source of inducible prostaglandin E_2_ production and mediates inflammatory symptoms including fever, loss of appetite and hyperalgesia. COX-1 is dispensable for fever, anorexia and hyperalgesia but is important for several other functions both under basal conditions and during inflammation. The differential functionality of the COX isoforms could be due to differences in the regulatory regions of the genes, leading to different expression patterns, or to differences in the coding sequence, resulting in distinct functional properties of the proteins. To study the molecular underpinnings of the functional differences between the two isoforms in the context of inflammatory symptoms, we used mice in which the coding sequence of COX-2 was replaced by the corresponding sequence of COX-1. In these mice, COX-1 mRNA was induced by inflammation but COX-1 protein expression did not fully mimic inflammation-induced COX-2 expression. Just like mice globally lacking COX-2, these mice showed a complete lack of fever and inflammation-induced anorexia as well as an impaired response to inflammatory pain. However, as previously reported, they displayed close to normal survival rates, which contrasts to the high fetal mortality in COX-2 knockout mice. This shows that the COX activity generated from the hybrid gene was strong enough to allow survival but not strong enough to mediate the inflammatory symptoms studied, making the line an interesting alternative to COX-2 knockouts for the study of inflammation. Our results also show that the functional differences between COX-1 and COX-2 in the context of inflammatory symptoms are not only dependent on the features of the promoter regions. Instead they indicate that there are fundamental differences between the isoforms at translational or posttranslational levels.

## Introduction

Prostaglandins comprise a large group of soluble lipid mediators, which fulfill a wide array of physiological functions. Prostaglandins are generated from arachidonic acid, a process which is mediated by cyclooxygenase enzymes. Specifically, most species have two cyclooxygenase enzymes (COX-1 and COX-2). Since the discovery of COX-2, a lot of research has dealt with the differential functionality of the two cyclooxygenases. Thus, COX-2 has been recognized as an immediate early gene with low basal expression and prompt up-regulation upon, for instance, inflammatory challenge, whereas COX-1 seems to exhibit a stable pattern of expression in most tissues [1]. To dissect the individual functions of the COX enzymes, several genetic and pharmacological tools have been developed. In 1995, two independent groups published the first data from global knockout (KO) strains of COX-2 [2, 3] and COX-1[4], showing that global knock out of COX-2 results in early development of kidney failure, spontaneous peritonitis, a substantial reduction of fetal survival related to failed closure of the ductus arteriosus, and several other severe defects, whereas global knock out of COX-1 renders generally healthy animals.

Despite poor breeding outcomes, COX-2 null mice have become important tools in studies of inflammation and cardiovascular medicine, as well as in cancer research. The use of global knockout animals, however, gives rise to questions of whether compensatory mechanisms come in to play. Thus, over the last decade, more specific genetic tools have been developed to aid in the study of COX enzyme isoform functionality and a conditional knockout allele of COX-2 allowing for tissue specific and inducible alterations in COX-2 expression is now available [5]. Also, a transgenic mouse line retaining peroxidase activity, but lacking the cyclooxygenase moiety of COX-2 (PGHS2 Y385F) has been described [6], as well as a transgenic mouse in which the COX-2 coding sequence has been exchanged for the coding sequence of COX-1 (COX-1>COX-2 animals) [7]. The purpose of the COX-1>COX-2 model was to elucidate to what extent COX-1 could fulfil the physiological functions of COX-2 when expressed in a similar pattern, and it was shown that this genetic interchange could ameliorate some aspects of COX-2 loss, such as a partial normalization of breeding outcomes. Administration of lipopolysaccharide (LPS) also resulted in up-regulation of COX-1 in macrophages indicating inducibility of the transgene [7].

The differential functionalities of COX-1 and COX-2 is also reflected in their involvement in systemic inflammatory symptoms. Although COX-1 is involved in the early phase of some responses to inflammation and in neuroinflammation [8–11], it is dispensable for systemic inflammatory symptoms such as fever, anorexia and hyperalgesia, which are instead dependent on COX-2 [12]. In fever, which is a highly conserved trait of acute inflammation, prostaglandin E2 is the critical prostanoid [13–18] and inhibition of prostaglandin synthesis is the main mechanism of action for common antipyretic drugs such as aspirin and paracetamol [14, 19, 20]. In acute inflammation, most of the PGE_2_ production is catalyzed by COX-2 [21] and mPGES-1 [22]. COX-2 is strongly up-regulated upon LPS stimulation and is necessary for a normal febrile response [12, 23, 24]. The central target region for fever-inducing PGE_2_ has been mapped to the anterior preoptic hypothalamus [25] and we have recently shown that the cerebral endothelium is the main source of COX-2 dependent PGE_2_ production in inflammatory fever [26]. Also in inflammation-induced anorexia, cyclooxygenase enzymes are known to be key players as COX inhibition results in attenuation of anorexia induced both by IL-1 beta [27] and LPS [28]. This effect was later shown to be specifically dependent on COX-2 [29].

The fact that many symptoms of systemic inflammation are dependent on COX-2 but not on COX-1, despite the fact that they both convert arachidonic acid to prostaglandin H_2_, could be due to differences in the regulatory regions of the genes, leading to different expression patterns. For example, the strong inflammation-induced expression of COX-2 in brain endothelial cells is critical for a normal febrile response [26], whereas COX-1 is not strongly induced in the brain in response to inflammation [1]. Alternatively, the critical feature that makes COX-2 pyrogenic and anorexigenic could be specific features of the coding sequence, leading to functional properties of the enzyme distinct to those of COX-1. Such properties could be critical for functional coupling to mPGES-1 or for the correct intracellular localization. To expand our knowledge of these aspects of isoform functionality, we investigated fever, anorexia and inflammatory pain in COX-1>COX-2 mice in which the coding sequence for COX-2 has been replaced with that of COX-1 [7].

## Results

### COX-1>COX-2 mice are born at almost the expected rate

The breeding outcome was comparable to what has been previously reported [7]. Although not reaching the theoretically expected 25% outcome of homozygous offspring from heterozygous (HZ) breeding, we noted a 15.7% outcome of live, homozygous transgenic offspring based on a total breeding cohort of 452 animals. This outcome is well above what is expected for global COX-2 KO animals [3]. Heterozygous animals were born at a frequency of 53.1%.

### COX-1>COX-2 mice show no inflammation-induced fever or loss of appetite

**Fig 1:**
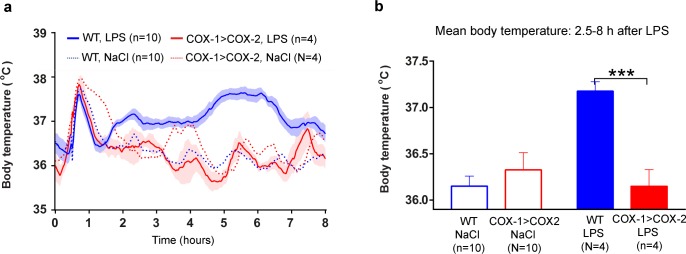
Attenuated febrile response in COX-1>COX-2 animals after administration of LPS. A. Telemetric recordings in freely moving animals showing the deep body temperature of COX-1>COX-2 animals or WT littermates after the administration of LPS (100 μg/kg) or vehicle intraperitoneally. B Average body temperature after LPS administration is completely normalized in COX-1>COX-2 animals compared to WT animals for the duration of the febrile response (2.5-8h post LPS). ***p<0,001

Upon intraperitoneal injection of lipopolysaccharide (LPS, serotype 0111:B4, 100 μg per kg), WT mice developed a classical, polyphasic inflammatory fever peaking at approximately 5 hour post injection (Fig 1A). COX-1>COX-2 mice failed to mount any febrile response as seen by the profile of the temperature graph. Mean fever during the period 2.5-8h was significantly attenuated (P = 0.0002) (Fig 1B). However, the initial hyperthermia caused by stress resulting from restraint and the injection procedure was intact (Fig 1A) in COX-1>COX-2 mice. See also **S1 Fig**.

**Fig 2:**
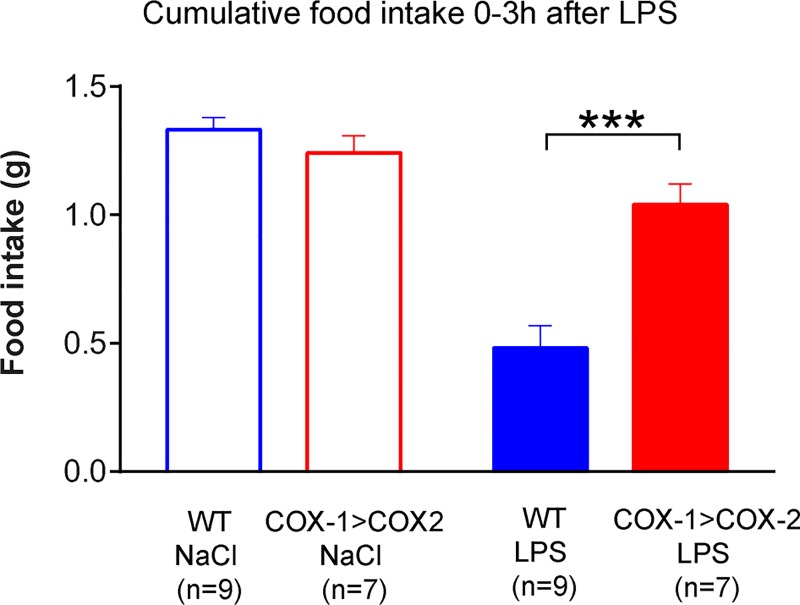
Cumulative food intake in COX-1>COX-2 and WT littermates during the first 3 hours after LPS injection (10μg/kg). The COX-1>COX-2 animals are significantly protected from the acute, anorexigenic effects of LPS (***p<0.001).

To investigate the extent of phenotype similarities with global COX-2 KO animals, we went on to investigate acute LPS-induced anorexia, another symptom of inflammation which is known to be COX-2 dependent [27–29]. Upon injection of LPS (10 μg per/kg), COX-1>COX-2 animals exhibited a level of food intake that was significantly higher than the food intake of WT littermates (p<0.0001) and approaching the level of food intake in WT littermates subjected to NaCl injections, showing that COX-1>COX-2 animals were protected from the anorexigenic effects of LPS (Fig 2). However, a strong anorectic response was seen in WT littermates injected with LPS (p< 0.001) as compared to NaCl injected littermates, indicating the validity of the model per se.

### COX-1>COX-2 mice display an attenuated response to formalin-induced pain

**Fig 3:**
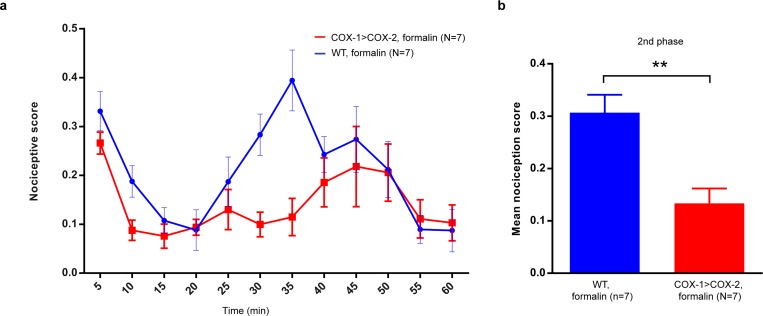
Quantification of the nociceptive response (flinching) to formalin-induced pain. (A) Nociceptive score was calculated for each 5 minutes during a total 60 minutes after subcutaneous injection of 2,5% formalin in the right hind paw. (B) The second phase of the nociceptive response was clearly attenuated in COX-1>COX-2 animals (**p<0.01).

To determine if the mutant mice behave abnormally also in response to a localized inflammatory insult, we next investigated nociceptive responses (flinching) to formalin-induced pain. The second phase of this response has previously been shown to partially depend on COX-2 [30]. We injected formalin in the dorsal surface of a back paw and monitored flinching behavior for one hour. The second phase of the nociceptive response was strongly attenuated in the mutant mice whereas the first phase was relatively intact (Fig 3A and 3B).

### COX-1 mRNA expression is induced by inflammation in COX-1>COX-2 mice

**Fig 4:**
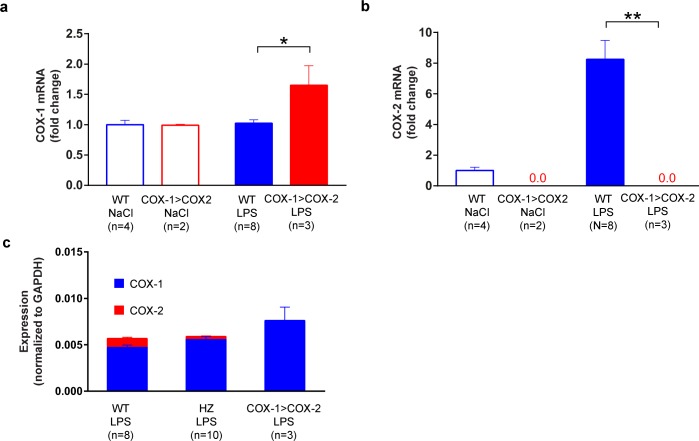
Quantification of COX-1 (A) and COX-2 (B) mRNA levels in hypothalami of COX-1>COX-2 animals and WT littermates 3 h after LPS injection (100μg/kg). A significant up-regulation of COX-1 is seen in COX-1>COX-2 animals (A). As expected, COX-2 was strongly induced in WT but not COX-1>COX-2 mice. The COX-1 expression signal in COX-1>COX-2 animals after LPS is at least as strong as the combined expression signal of COX-1 and COX-2 in WT animals after LPS, indicating that the induction of COX-1 at least corresponds to the increase in COX-2 mRNA in absolute terms (C). COX-1>COX-2 heterozygous (HZ) animals were included as comparison. (*p<0.05; **p<0.01)

Next, we investigated if COX-1>COX-2 mice displayed an up-regulation of COX-1 in brain structures in which COX-2 levels normally rise upon inflammatory stimulation. Thus, we went on to investigate the gene expression levels of COX-1 and COX-2. We chose to quantify the gene expression levels in hypothalamic tissue from animals injected with LPS 3h prior to dissection, since this is a well-established time point of strong changes in the expression of inflammatory genes [26]. As expected, COX-1 mRNA levels were not elevated in response to LPS in WT mice. In contrast, we could detect a significant (approximately 62%) up-regulation of COX-1 in COX-1>COX-2 animals upon LPS administration (p = 0.0142) (Fig 4A). In WT animals, COX-2 was up-regulated approximately 720% (p = 0.0026) after LPS-administration, whereas no COX-2 mRNA was detected in COX-1>COX-2 animals (Fig 4B). After LPS injection, COX-2 mRNA was detected at around cycle 29 and COX-1 mRNA at around 26.5 in WT mice. This strongly indicates that COX1 mRNA is expressed at much higher levels than COX-2 mRNA in the hypothalamus both under basal and inflammatory conditions. Thus, the COX-1 expression signal in COX-1>COX-2 animals after LPS is at least as strong as the combined expression signal of COX-1 and COX-2 in WT animals after LPS. This indicates that COX-1 induction in the COX-1>COX-2 mice at least matches the corresponding induction of COX-2 mRNA in WT animals in absolute terms (Fig 4C). COX-1>COX-2 heterozygous animals (HZ) were included as comparison (Fig 4C).

Collectively, this shows that COX-1 mRNA is induced by inflammation in COX-1>COX-2 mice. The fact that the fold-change of the induction of COX-1 in COX-1>COX-2 mice was lower than the corresponding COX-2 induction in WT mice might be explained by the higher COX-1 expression at the basal state.

### COX-1 protein is not expressed in the expected pattern in COX-1>COX-2 mice

**Fig 5:**
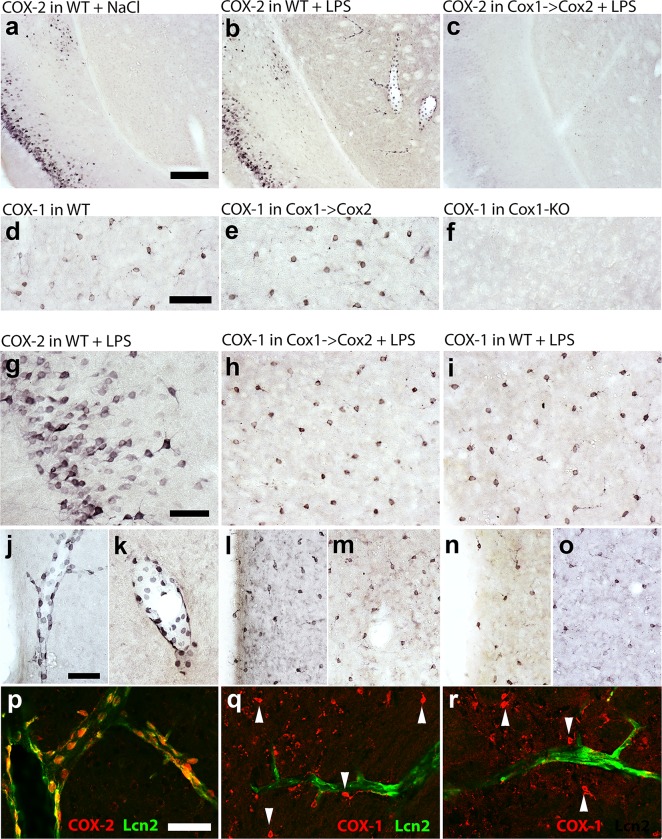
Photomicrographs showing the COX-1/COX-2 expression pattern in the brain 3 hours after intraperitoneal injection of NaCl or LPS (100 μg/kg). Basal COX-2 expression in WT animals was prominent in parts of the cerebral cortex (a) whereas, in WT mice treated with LPS, strong COX-2 expression was seen also in cells with endothelial morphology lining cerebral vessels (b). No COX-2 could be detected in brains from COX-1>COX-2 mice treated with LPS (c). Strong COX-1 labeling was found in cells with microglial morphology in both WT and COX-1>COX-2 mice (d, e; cerebral cortex). Brain sections from mice lacking COX-1 displayed no COX-1 labeling (f). Neuronal COX-2 expression was seen in the cerebral cortex of WT mice (g) injected with LPS, but no corresponding expression pattern of COX-1 was seen in COX-1>COX-2 mice (h). Instead, only cells with microglial morphology expressed COX-1 in the corresponding region of the brain in COX-1>COX-2 mice (h) in a pattern identical to COX-1 in WT mice (i). Vascular COX-2 expression in LPS-treated WT mice (j, k) was compared to COX-1 expression in COX-1>COX-2 mice (l, m) and COX-1 expression in WT animals (n, o). Neither in the hypothalamus (j, l, n) nor elsewhere (k, m, o; striatum), the COX-1 expression in COX-1>COX-2 mice matched that of COX-2 in WT mice. Dual labeling analysis and confocal microscopy showed that activated endothelial cells, identified by detection of Lcn2 expression, expressed COX-2 in WT mice (p) but that no corresponding COX-1 expression could be seen in activated endothelial cells in COX-1>COX-2 mice (q, r). Scale bar (same for all figures within a given row) in a = 200 μm, d, g, j and p = 50 μm.

We proceeded to investigate if COX-1 protein was expressed in a COX-2-like pattern in the COX-1>COX-2 mice. Using immunohistochemistry, we first investigated the Cox-2 expression pattern in the brain 3 hours after intraperitoneal injection of NaCl or LPS (100 μg/kg). As expected, COX-2 was expressed in neurons of several structures under basal conditions. For example, COX-2 expression was prominent in parts of the cerebral cortex (Fig 5A). In mice treated with LPS, strong COX-2 expression was seen also in cells with endothelial morphology lining cerebral vessels (Fig 5B). We could detect no COX-2 in brains from COX-1>COX-2 mice (Fig 5C). Next we investigated the COX-1 expression in WT and COX-1>COX-2 mice. Strong labeling was found in cells with microglial morphology in both WT and COX-1>COX-2 mice (Fig 5D and 5E). Brain sections from mice lacking COX-1 displayed no COX-1 labeling (Fig 5F) indicating that the immunohistochemistry was specific. Next we investigated if COX-1>COX-2 mice expressed COX-1 in a COX-2-pattern as would be expected. Since a strong neuronal COX-2 expression was seen in the cerebral cortex of WT mice (Fig 5G), we expected COX-1 expression in the same cells in COX-1>COX-2 mice. Surprisingly, no such expression was seen (Fig 5H). Instead, only cells with microglial morphology expressed COX-1 in the corresponding region of the brain in COX-1>COX-2 mice (Fig 5H) in a pattern identical to COX-1 in WT mice (Fig 5I). Since the neuronal COX-2 expression is constitutive, the lack of labeling could not be due to different kinetics in the induction of COX-1 and COX-2. Further, the vascular COX-2 expression in LPS-treated WT mice was not matched by a corresponding COX-1 induction in COX-1>COX-2 mice (Fig 5J–5O). In line with this, dual labeling analysis and confocal microscopy showed that activated endothelial cells, identified by detection of Lcn2 expression, expressed COX-2 in WT mice (Fig 5P) but that no corresponding COX-1 expression could be seen in activated endothelial cells in COX-1>COX-2 mice (Fig 5Q and 5R). Collectively, the immunohistochemical analysis strongly indicates that the COX-1 expression in COX-1>COX-2 mice is very similar to the COX-1 expression in WT mice and that there is no additional expression with a COX-2-like pattern.

## Discussion

Here we show that mice in which the coding sequence of COX-2 has been replaced by the coding sequence of COX-1 exhibit several of the physiological traits of global COX-2 KO animals in the setting of acute inflammation, whereas offspring yield is improved considerably compared to global COX-2 KOs. This is a potentially useful finding, since breeding of COX-2 KOs requires breeding of large cohorts of heterozygous animals and is therefore highly time and resource consuming. Our results also show that the distinct functions of the COX isoforms in the process underlying inflammatory symptoms are not only explained by features of the promoter regions.

Our results demonstrate that COX-1 expressed under the COX-2 promoter can substitute for some COX-2 functions but not all. The fact that both inflammation-induced fever and anorexia of COX-1>COX-2 animals are blocked in a manner comparable to what is seen in COX-2 KO animals or after pharmacological COX-2 inhibition [12, 27–29], indicate that the COX-2 dependent components of brain mediated inflammatory symptoms are blocked in COX-1>COX-2. This is further underpinned by the observation that COX-2 dependent nociception is also reduced in these animals. In line with this observation, COX-1>COX-2 animals are not protected against all abnormalities seen in global COX-2 KO animals, since they are prone to the development of spontaneous peritonitis [7]. Thus, even if the usability of COX-1>COX-2 animals as a substitute of global COX-2 KO animals will have to be specifically determined for other lines of study, the data collected so far indicate that they are very similar to COX-2 KOs in many respects, with fetal survival as an important exception. The rescue of fetal survival in COX-1>COX-2 animals might be an effect of the transgene driving a small amount of COX-2 promoter dependent prostaglandin production. If so, even very low levels of PGE_2_ in the right tissues at the right time points are sufficient to ameliorate some of the defects resulting from total lack of COX-2 enzyme activity, such as fetal survival and kidney pathology [7]. The hypothesis that very low levels of PGE_2_ synthesis capacity is enough for normal fetal survival whereas high levels are needed for the generation of fever is further supported by the fact that mice heterozygous for COX-2 display a markedly attenuated febrile response [14], but are born at the expected ratio.

Our molecular analysis shows that COX-1 mRNA is induced by inflammation in COX-1>COX-2 animals but not in WT mice. This induction did not reach the same fold change magnitude as the corresponding COX-2 mRNA induction. However, since the basal levels of COX-1 mRNA are much higher than the corresponding COX-2 mRNA levels this is what could be expected even if the transgene worked perfectly on the transcriptional level. This is illustrated by the fact that the increase in COX-1 expression signal in COX-1>COX-2 animals after LPS is at least as strong as the combined increase in expression signal of COX-1 and COX-2 in WT animals after LPS. Although this interpretation is complicated by the fact that mRNA stability of COX-2 is lower than that of COX-1 in most systems [31, 32], and a potential difference in qPCR detection efficiency between the genes, it indicates that the transgene works as expected in terms of mRNA-expression.

In contrast, our histological data indicate that the COX-1 protein induction in the brain endothelium is absent or at least very much lower than the COX-2 induction it should mimic. In principle, this could be due to lower sensitivity of the COX-1 immunohistochemistry compared to the COX-2 immunohistochemistry. However, we find this unlikely since we could detect a robust COX-1 expression in microglial cells in WT and mutant mice. The lack of obvious induction is surprising since COX-1 protein levels are induced in macrophages from COX-1>COX-2 animals treated with LPS [7]. One possible explanation for the low (or absent) induction is that increased expression of COX-1 in cells normally only expressing low levels of COX-1 is counteracted by prompt degradation of the protein. Further, several pathways are known to be active in COX-2 protein degradation [32] and, for instance, increased degradation due to lack of some post-translational modification may cause the difference in protein levels. Other attempts at altering the expression of cyclooxygenase enzymes have also rendered surprising data. Thus, in an attempt to express COX-2 under the promoter of COX-1, a non-functional transgene has been reported [33]. It was suggested that an alteration of a signaling peptide involved in protein processing might have caused aberrant protein expression [33]. Since a similar signal peptide of COX-1 is known to be altered in the COX-1>COX-2 transgene [7], such a mechanism could potentially explain the partly surprising findings presented in this study.

In summary, we demonstrate that exchange of the coding sequence of the COX-2 gene to that of COX-1 made the gene unable to fulfill its function in the generation several brain-mediated symptoms of acute inflammation. Since the high fetal mortality associated with deletion of COX-2 was not observed in mice carrying the hybrid gene, although they copy the phenotype of COX-2 knockouts in brain-mediated inflammatory symptoms, these mice are interesting alternatives to COX-2 knockouts for the study of inflammation. Collectively, this shows that the different patterns of mRNA expression resulting from differences in the promoter regions cannot fully explain why COX-1 and COX-2 are involved in so different biological functions.

## Methods

### Animals

All animal experiments were conducted with approval of the local animal care and use committee of Linköping (permit number 60–09) and in strict compliance with international guidelines.

We used a mouse line expressing *Ptgs1* gene under the *Ptgs2* gene regulatory elements (*Ptgs2^tm2.1(Ptgs1)Fun^*, known as “COX-1>COX-2”) [7]. The line was obtained from Jackson Laboratories (Bar Harbour, ME) and bred in our animal facility to generate homozygous mutants and wild type littermates as experimental animals. Mice were genotyped by PCR (forward primer: ACC TCT GCG ATG CTC TTC C, reverse primers: ACT GGT CAA ATC CTG TGC TC and CTC ACA TTG GAG AAG GAC TCC). Animals were housed in 12 hour light-dark cycle (lights on 07:00) with ad libitum access to chow and water.

#### Intraperitoneal injections of LPS

Animals were injected with *Escherichia coli* lipopolysaccharide (LPS) of serotype O111:B4 (Sigma-Aldrich). In fever experiments, qPCR and immunohistochemistry a dose of 100 μg/kg was used, whereas a dose of 10 μg/kg was used in food intake experiments. The LPS was diluted in 100μl sterile 0.9% NaCl solution and injected intraperitoneally 1.5 h after lights on in fever experiments and 1h before lights off in food intake experiments. A similar volume of vehicle was given to the control group. These doses of LPS are known to induce a strong febrile response without substantial hypothermia and a reproducible anorexia respectively [13, 26, 34].

#### Surgical procedures

Animals were provided preoperative and postoperative analgesia with buprenorphine (25μg/kg; Temgesic, RB Pharmaceuticals). Animals were anaesthetized by 4% isoflurane (Abbot) in 100% O_2_ in an induction cage. Anesthesia was maintained with 1.5% isoflurane in 100% O_2_ on a face mask. Telemetry transmitters (Data Sciences international) were implanted by a small incision at the abdominal midline and peritoneum and skin was sutured in layers. Animals were allowed to recover for one week postoperatively.

#### Measurements of fever

Continuous telemetry with an intra-abdominal transmitter in freely moving animals was used to record body temperature (transmitter and recording system: TA11TAF10, Data Sciences International). Basal temperature was recorded for 24 h before any experimentation. From the surgery to the end of the experiment, mice were kept in a thermoneutral environment (29°C).

#### Measurements of food intake

Animals were housed one to a cage one week prior to LPS injection. One hour before the start of the dark cycle, mice were injected with LPS (10 μg/kg) or vehicle. Food was returned after 1 h and food intake was recorded 3h into the dark cycle according to a procedure previously described [35].

#### Formalin nociceptive scoring

Animals were kept in a transparent plexiglass box 20 (w) x 15 (d) x 25 (h) cm for habituation for 30 minutes. Diluted formalin (2.5%, 20μl) was injected subcutaneously on the dorsal aspect of the right hind paw and the behavior of the mouse was recorded for 60 minutes using a Canon LEGRIA HF R48 video camera. The times spent by the mouse in licking, biting and shaking of formalin injected paw were measured based on the video recordings. The nociceptive score was calculated for each 5 minutes (300 seconds) of total 60 minutes and represented on a rating scale [30].

### qPCR measurements

Mice were subjected to intraperitoneal injection of LPS (100 μg/kg) or vehicle and euthanized by CO_2_ asphyxiation after 3 h. Animals were transcardially perfused with sterile saline solution to remove blood and hypothalamus tissue was dissected in accordance with a previously described procedure [36]. Tissue was preserved in RNA later solution (Qiagen) at -70 C until RNA extraction, which was done using RNeasy Lipid Mini kit (Qiagen). Reverse transcription was performed with High Capacity cDNA Reverse Transcription kit (Applie Biosystems) and qPCR was conducted with Gene Expression Master Mix (Applied Biosystems) on 96 well plates (7900HT Fast RT-PCR system; Applied Biosystems). The following assays were used: *Ptgs1*: Mm00477214_m1 *Ptgs2*: Mm00478374_m1 and GPDH (as reference gene): Mm99999915_g1. The levels of COX-1 and COX-2 mRNA were normalized to the levels of GAPDH using the Delta Delta CT method [37].

### Immunohistochemistry

Mice were euthanized by CO_2_ asphyxiation 3h post LPS or saline administration and perfused transcardially with saline (0.9%), followed by buffered paraformaldehyde-solution (4%, pH 7.4). Brains were post-fixed for 3h and then incubated in 30% sucrose-PBS solution overnight. 40 μm thick coronal sections were cut on a freezing microtome (Leica Biosystems). Immunolabeling for Cox-1 or Cox-2 was performed on free-floating sections using avidin-biotin-HRP system with 3, 3’-diaminobenzidine tetrahydrochloride (DAB; Sigma) as chromogen. Briefly, the sections were first incubated in either goat anti-Cox-1 (G1752, 1:1000; Santa Cruz Biotechnology, Santa Cruz, CA) or rabbit anti-Cox-2 (R1747, 1:1000; Santa Cruz Biotechnology) overnight at room temperature, followed by biotinylated horse anti-goat IgG (1:1000; Vector Labs, Burlingame, CA) or goat anti-rabbit IgG (1:1000; Vector Labs), for 2h at room temperature. Sections were then incubated in avidin-biotin complexes (1:1000; Vector Labs) for 2h and color was then developed using DAB containing H_2_O_2_ (0.01%) and nickel ammonium sulfate (2.25%) in sodium- acetate buffer (0.1 M, pH 6.0)for 6 min, according to standard protocol [38].

For immunofluorescent double-staining, sections were first incubated for 45 min in blocking solution (1% BSA and 0.3% Triton X-100 in PBS) and then overnight in a mixture of antibodies: rabbit anti-Cox-1 (160109, Cayman) plus goat anti-Lipocalin-2 (AF1857, R&D Systems) or rabbit anti-Cox-2 (sc-1747R, Santa Cruz Biotechnology) plus goat anti-Lipocalin-2 (AF1857, R&D Systems). After rinsing in PBS, sections were incubated for 2h with secondary antibodies: donkey anti-rabbit Alexa Fluor 568 and donkey anti-goat Alexa Fluor 488. All primary and secondary antibodies were diluted 1:500. All steps were performed in room temperature. Double-labeled images were obtained by sequential scanning.

### Statistical analysis

Experiments comprising four groups were conducted in 2x2 factorial design and analyzed by two-way ANOVA and Tukey’s post hoc test for multiple comparisons. P values of <0.05 were considered significant.

## Supporting Information

S1 Fig Fever and mean fever, PLOS ONE version.pzfx. Underlying temperature measurements for **Fig 1**.
(PZFX)
